# Neutron Scattering of Aromatic and Aliphatic Liquids

**DOI:** 10.1002/cphc.201600149

**Published:** 2016-04-13

**Authors:** Marta Falkowska, Daniel T. Bowron, Haresh G. Manyar, Christopher Hardacre, Tristan G. A. Youngs

**Affiliations:** ^1^ STFC ISIS FacilityRutherford Appleton Laboratory, HarwellOxford, Didcot, OxonOX11 0QXUK; ^2^ CenTACatSchool of Chemistry and Chemical EngineeringQueen's UniversityBelfastStranmillis RoadBT9 5AGUK

**Keywords:** aliphatic liquids, aromatic liquids, liquid structure, liquids, neutron diffraction

## Abstract

Organic solvents, such as cyclohexane, cyclohexene, methylcyclohexane, benzene and toluene, are widely used as both reagents and solvents in industrial processes. Despite the ubiquity of these liquids, the local structures that govern the chemical properties have not been studied extensively. Herein, we report neutron diffraction measurements on liquid cyclohexane, cyclohexene, methylcyclohexane, benzene and toluene at 298 K to obtain a detailed description of the local structure in these compounds. The radial distribution functions of the centres of the molecules, as well as the partial distribution functions for the double bond for cyclohexene and methyl group for methylcyclohexane and toluene have been calculated. Additionally, probability density functions and angular radial distribution functions were extracted to provide a full description of the local structure within the chosen liquids. Structural motifs are discussed and compared for all liquids, referring specifically to the functional group and aromaticity present in the different liquids.

## 1 Introduction

The industrial importance of solvents is evident by the fact that in 2013 about US$ 25 billion were generated from their worldwide sale.^1^ Furthermore, the global solvents market value has been predicted to increase by 4 % per year until 2021, owing to the demand for them in the developing countries in the Asia‐Pacific region.^1^ Solvents have a wide range of uses and common applications, for example, in paints, varnishes, printing inks, adhesives, pharmaceuticals, cosmetics and cleaners. Moreover, solvents play a significant role in industrial production processes, for example, in chemical syntheses, purification processes, environmental health and safety actions. Examples of the roles of solvents include transport media for heat or products of the reaction, changing the reaction mechanism, reducing the concentration of aggressive compounds, and more. The most common groups of compounds used as conventional solvents are aromatic hydrocarbons, aliphatic hydrocarbons and alcohols.

Despite the fact that liquid solvents are disordered materials, it is possible to distinguish their local structure by using the atomic pair distribution function, which describes the likelihood of finding a second atom at a given separation from one placed at the origin.^2^ Neutron diffraction has proven to be an ideal method for structural studies on ionic liquids,^3^, ^4^, ^5^ atomic liquids,^6^ molecular liquids such as water,^7^ methanol,^8^ ethanol,^9^ tertiary butanol,^10^ 2,2,2‐trifluoroethanol,^11^ benzene and toluene,^12^ nitromethane,^13^ and methylene chloride^14^ as well as liquid mixtures such as methanol/water,^15^ isopropanol/water,^16^ tert‐butyl alcohol/water,^17^ and tert‐butyl alcohol/cyclohexene/water.^18^ In spite of the availability of neutron techniques able to give insight into the underlying correlations that govern the chemical properties of these ubiquitous solvents, the structures of many other important liquids have yet to be investigated by this method.

Cyclohexane is one of the most widely used solvents, owing to its higher density, viscosity, boiling and crystallisation temperatures compared with other C6 alkanes. This molecule has two stable conformers, the chair and the twist‐boat,^19^ of which the chair form is lower in energy. The structure of cyclohexane in the liquid state has been studied by using molecular dynamics^20^, ^21^ as well as neutron^22^ and X‐ray^23^, ^24^ diffraction studies. In contrast, the liquid structure of cyclohexene has not been investigated as widely as cyclohexane, despite the fact that it is frequently used in industry as a solvent and a reagent in, for example, adipic acid production. The presence of one double bond in the molecule flattens it and, as a consequence, the chair conformation is replaced by a half‐chair form.^25^ Methylcyclohexane is used as a solvent and is a component of jet fuel; it has two conformers with the methyl group either in an axial or equatorial configuration. In this system, the latter conformation is higher in energy, resulting in a ratio of 20:1 of equatorial/axial conformers at 25 °C.^19^ This liquid has been studied by using X‐ray diffraction^26^ and computational techniques.^27^ Benzene, in turn, can be treated as a prototypical molecular liquid, from which the π–π interactions in more sophisticated systems can be evaluated.^12^ Owing to its scientific and industrial significance, benzene has been investigated extensively through Monte Carlo simulations,^28^, ^29^ molecular dynamics,^30^, ^31^ as well as X‐ray and neutron diffraction.^12^, ^31^, ^32^, ^33^, ^34^ Four possible interaction geometries between benzene molecules have been reported, that is, the sandwich, T‐shape, parallel displaced, and Y‐shape, of which the latter two are preferred.^12^ Toluene, which is a less carcinogenic solvent alternative to benzene, has also been studied by using various methods.^12^, ^31^, ^32^, ^34^, ^35^

Herein, we apply neutron scattering with isotopic substitution^36^ to examine the influence of the presence of a double bond (cyclohexene) and a functional group (methylcyclohexane) on the orientational organisation compared with the liquid structure of cyclohexane. The liquid structures of benzene and toluene have also been measured and compared to previous literature results as well as the other systems in the present study to probe the influence of the presence of the methyl group across aromatic and aliphatic systems.

Data were collected on the Near‐ and InterMediate‐Range Order Diffractometer (NIMROD) at the ISIS Facility at STFC Rutherford Appleton Laboratory, Harwell Campus, Oxfordshire, UK.^37^ A full account of the experimental method is provided in the Supporting Information. The results obtained for the five systems investigated, that is, cyclohexane, cyclohexene, methylcyclohexane, benzene and toluene, are described individually and, in the last section, a comparison of local ordering in these liquids is conducted.

## 2 Results and Discussion

**Figure 1:**
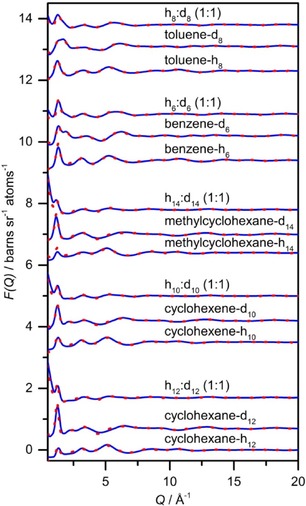
Experimental (red dotted lines) and EPSR‐fitted (blue solid lines) interference differential cross sections as a function of *Q* for different isotopically substituted liquids, that is, cyclohexane, cyclohexene, methylcyclohexane, benzene and toluene.

**Figure 2:**
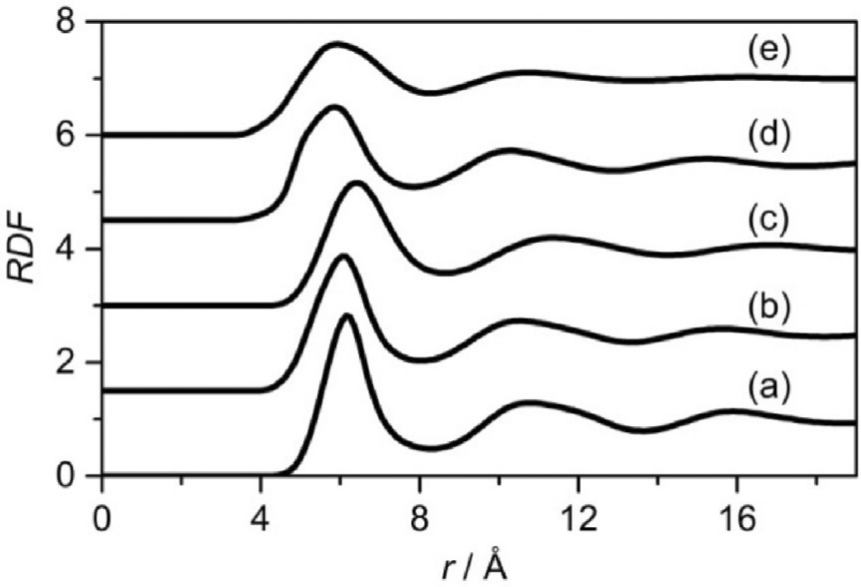
Molecular centre radial distribution functions for a) cyclohexane, b) cyclohexene, c) methylcyclohexane, d) benzene and e) toluene.

**Figure 3:**
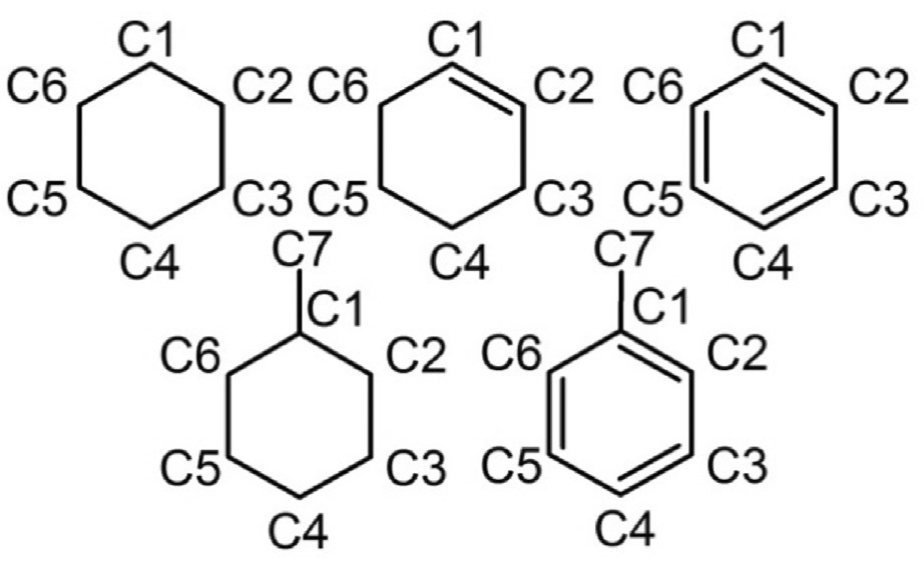
Carbon numbering for cyclohexane, cyclohexene, methylcyclohexane, benzene and toluene used throughout the paper.

Neutron diffraction data and EPSR fits for neat cyclohexane, cyclohexene, methylcyclohexane, benzene and toluene are shown in Figure 1. Excellent agreement was obtained between the experimental data and the EPSR‐derived structure factors in all cases. The small residual disagreement at lower *Q* values is largely attributed to errors in the subtraction of inelasticity effects from the data, which is confirmed by the slightly better agreement obtained for deuterated samples, in which these effects are less striking. The simulation boxes described by the EPSR‐derived structure factors were used to calculate radial distribution functions (RDFs), partial radial distribution functions (PRDFs), angular radial distribution functions (ARDFs) and spatial distribution functions (SDFs). Figure 2 shows the RDFs between the molecular centres of geometry for cyclohexane, cyclohexene, methylcyclohexane, benzene and toluene, and the coordination numbers for the first coordination shell were calculated for each compound by integrating the area underneath the first RDF peak (Table 1). Figure 3 shows the numbering of carbon atoms in the molecule for each studied liquid, which is used throughout this work.

**Table 1:** Coordination shell populations determined from molecular centre radial distribution functions for cyclohexane, cyclohexene, methylcyclohexane, benzene and toluene. Coordination numbers were calculated by integration of the relevant RDF up to the position of the first minimum.

Compound	Position of 1^st^ shells maximum [Å]	Position of 2^nd^ shell maximum [Å]	Relative position of 2^nd^ shell maximum [Å]	1^st^ shell cut‐off [Å]	1^st^ shell coordination number
cyclohexane	6.2	10.8	1.74	8.3	12.8
cyclohexene	6.1	10.6	1.74	8.1	12.8
methylcyclohexane	6.5	11.4	1.75	8.7	12.6
benzene	5.9	10.3	1.75	7.9	13.4
toluene	6.0	10.8	1.80	8.3	12.9

### 2.1 Cyclohexane

For cyclohexane, three distinct coordination shells (Figure 2) can be identified in the RDF, which is in good agreement with both X‐ray studies^21^ as well as other neutron diffraction studies.^22^ In cyclohexane, all peaks are sharp and well‐resolved, which indicates that the molecules occupy well‐defined positions in both relative orientation and in distance. The first coordination shell for cyclohexane is localised at a distance of 6.2 Å, with the RDF having a peak height of 2.8, which indicates that it is almost three times more likely that two molecules would be found at this separation. The first coordination shell contains a population of 12.8 molecules, which are the immediate neighbours of the central molecule. The maximum of the second coordination shell is at a distance of 10.8 Å, which is 1.74 times further than that of the first coordination shell. Gontrani et al.^21^ employed X‐ray diffraction to study the liquid structure of cyclohexane and found the first maximum to be at 6.25 Å, and the second at 11.5 Å, which is in good agreement with data presented in our work. Similarly, Katayama et al.^24^ reported that the first coordination shell is placed within a distance of 5.0–8.0 Å.

**Figure 4:**
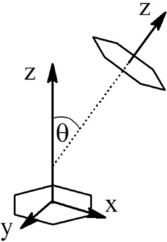
Definition of the angle *θ* between the *z* axis of the central and surrounding molecules used for calculation of angular radial distribution functions. The angle *θ*=90±10° corresponds to molecules that are perpendicular, whereas *θ*=0±10° defines two parallel molecules.

The orientation of molecules within a liquid can be studied by analysing the ARDF, that is, the RDF plotted as a function of the angle *θ* between the *z* axes of the central and surrounding molecules (0°<*θ*<90°). For all liquids studied, herein, the *z* axis is defined as pointing out of the plane of the ring, while the *x* and *y* axes lie in the plane of the ring (Figure 4).

**Figure 5:**
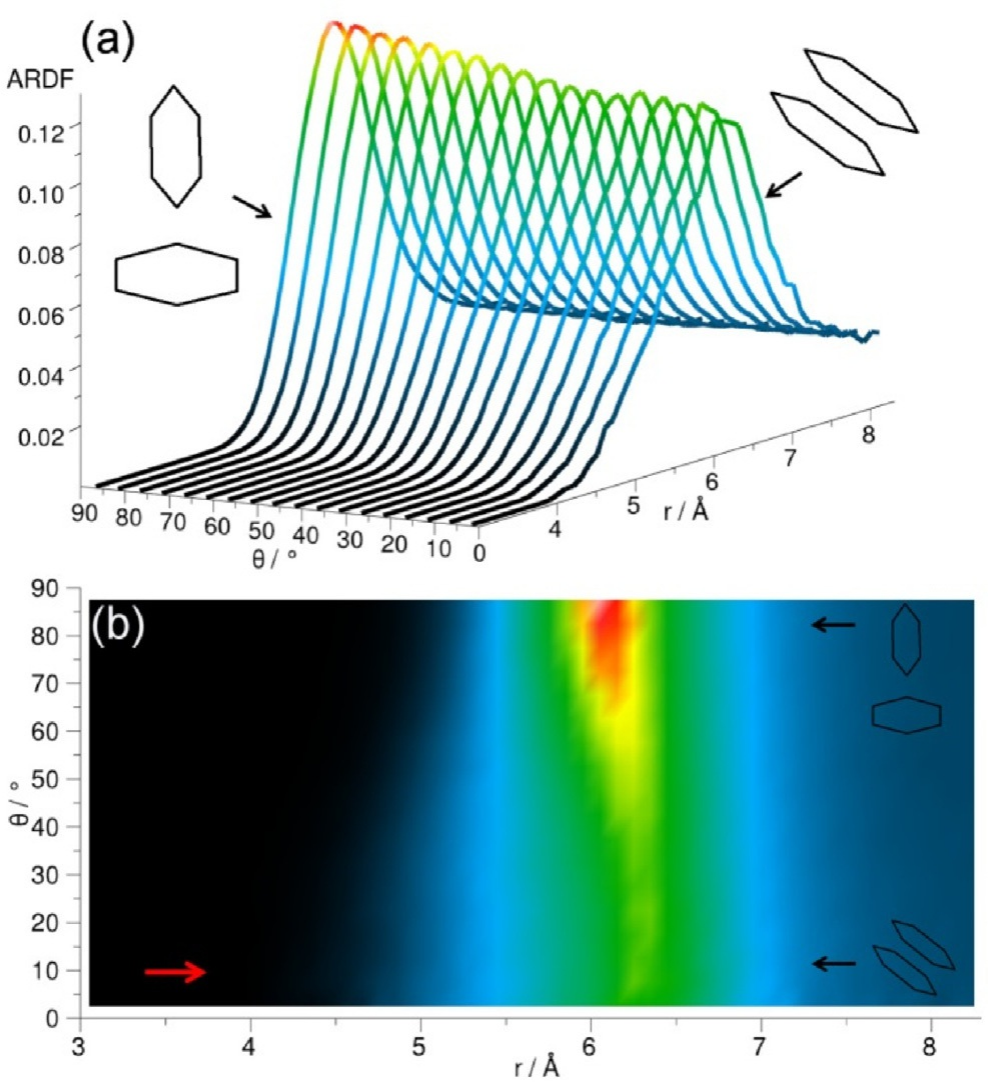
Angular radial distribution function for cyclohexane calculated as a function of the angle between the *z* axes of the central and surrounding molecules (0°<*θ*<90°), which in the studied liquid has been defined as shown in Figure 4. Plot (b) is the overhead projection of the 3D plot (a). The red arrow in plot (b) indicates a small increase in ARDF, which signifies a slight preference of the parallel orientation at distances shorter than 5.45 Å.

The ARDF for cyclohexane shows the highest peak within the distance range, corresponding to the first RDF coordination shell for the perpendicular arrangement of surrounding molecules (Figure 5). However, at distances shorter than 5.45 Å, a slight preference of the parallel orientation is observed, as shown by the small increase in intensity at this distance in Figure 5 b. Similar preference for the plane parallel arrangement of molecules occupying positions at shorter distances was found in Ref. 23.

**Figure 6:**
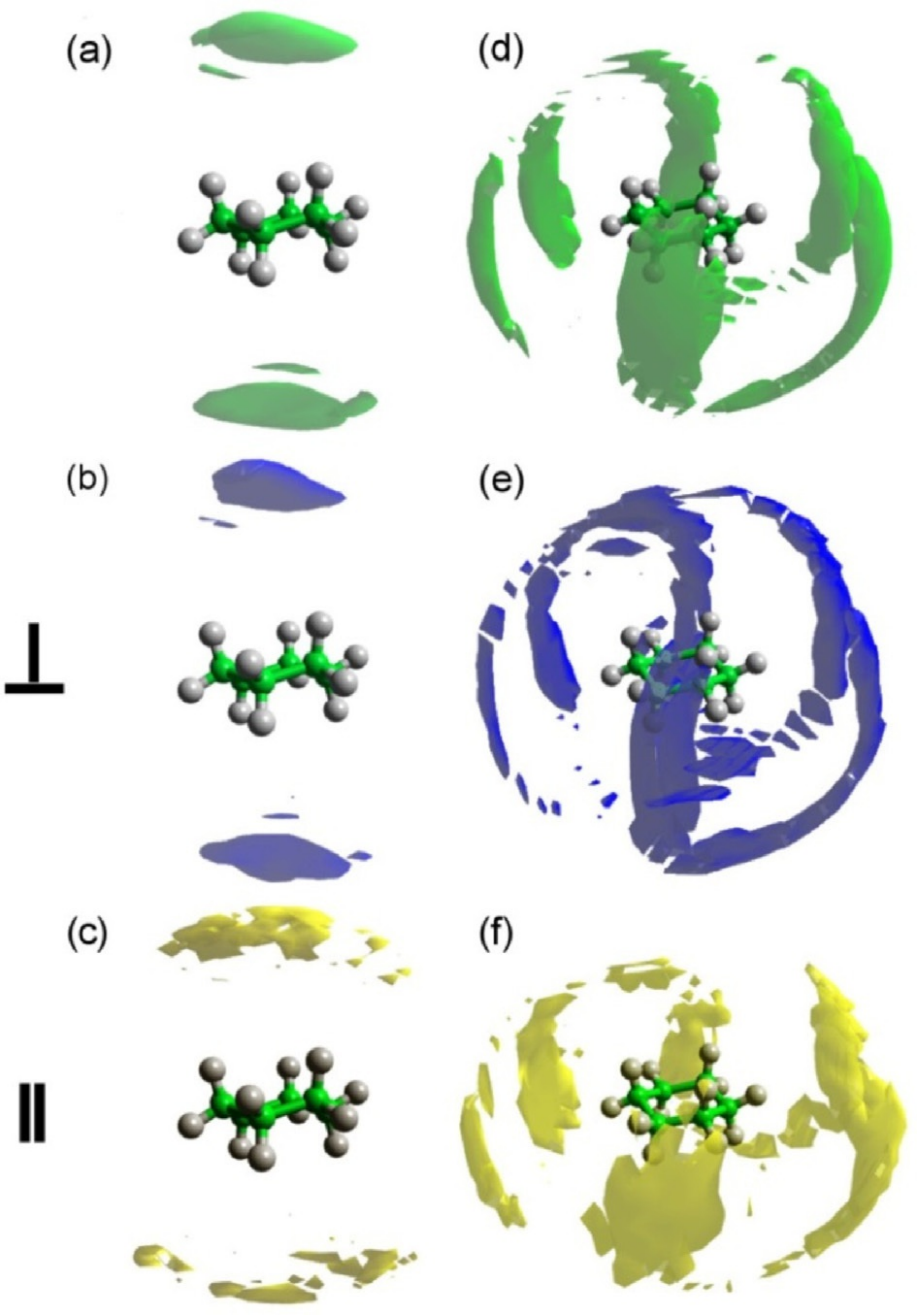
Spatial probability densities for liquid cyclohexane calculated within two distances ranges determined from the ARDF, that is, 0–5.45 Å and 5.45–8.30 Å from the central molecule. The functions represent the top 20 % of a) all molecules, b) perpendicular molecules only (*θ*=90±10°), and c) parallel molecules only (*θ*=0±10°) with respect to the central molecule found within *r*=0–5.45 Å. Additionally, functions representing the top 10 % of d) all molecules, e) perpendicular molecules only (*θ*=90±10°), and f) parallel molecules only (*θ*=0±10°) with respect to the central molecule found within *r*=5.45–8.30 Å are shown. Surfaces were plotted using the Aten software.^38^

Detailed information concerning the positions occupied by the surrounding cyclohexane molecules can be achieved by plotting the spatial probability densities (Figure 6). They represent the most probable positions for surrounding molecules within the specified distance from the central molecule and can also be calculated for specific orientations of surrounding molecules (as defined in Figure 4).

At distances shorter than 5.45 Å, molecules occupy positions above and below the ring, which is often referred to as a sandwich arrangement for parallel molecules. While analysing the most likely positions within this range with respect to the molecules′ orientation, it can be seen that parallel‐oriented molecules approach the central molecule more closely than perpendicularly oriented molecules, as expected from steric considerations. Only 2 % of all molecules within the distance range shorter than 5.45 Å are parallel‐oriented molecules, whereas 12 % of all molecules are arranged perpendicularly. At distances within the first coordination shell, but longer than 5.45 Å, an approximate six‐fold symmetric distribution of cyclohexane molecules is observed, with the most probable positions being perpendicular to the middle of C−C bonds of the central molecule. Analysis of the most likely positions for parallel and perpendicularly oriented surrounding molecules reveals that the former are at longer separations from the central molecule than the latter, as expected. As found for shorter distances, fewer molecules (1.5 %) within the range 5.45–8.30 Å are parallel, compared with 18 % being perpendicular. The low amount of parallel molecules that contribute to the first coordination shell region thus decrease the statistics, and hence the definition and smoothness of the plotted density function are reduced. In contrast to our findings, Bochynski and Drozdowski,^23^ who studied cyclohexane by using X‐ray diffraction, identified a large (46 %) preference for parallel orientation.

### 2.2 Cyclohexene

**Figure 7:**
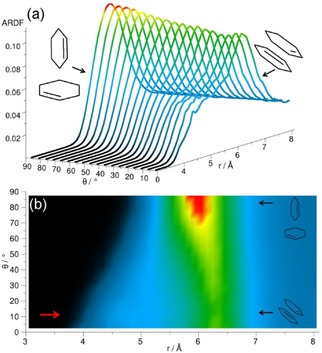
Angular radial distribution function for cyclohexene calculated as a function of the angle between the *z* axes of the central and surrounding molecules (0°<*θ*<90°), which in the studied liquid has been defined as shown in Figure 4. Plot (b) is the overhead projection of the 3D plot (a). The red arrow in plot (b) indicates a small increase in ARDF, which signifies a slight preference of the parallel orientation at distances shorter than 5.25 Å.

The centre of geometry RDF for cyclohexene reveals three distinct coordination shells (Figure 2), the first of which has a maximum value of 2.4 at a distance of 6.1 Å from the central molecule and contains 12.8 neighbouring molecules. The second maximum is at a distance of 10.6 Å, which is 1.74 times longer than the first maximum. The ARDF for cyclohexene reveals the highest peak corresponding to the perpendicular orientation (*θ*=90±10°) preferred at distances greater than 5.25 Å, but also parallel molecules (*θ*=0±10°) have a significant probability of being found within this range (Figure 7). Additionally, the shoulder at shorter distances (less than 5.25 Å) can be observed, showing a slight preference for parallel orientation of surrounding molecules in this range.

**Figure 8:**
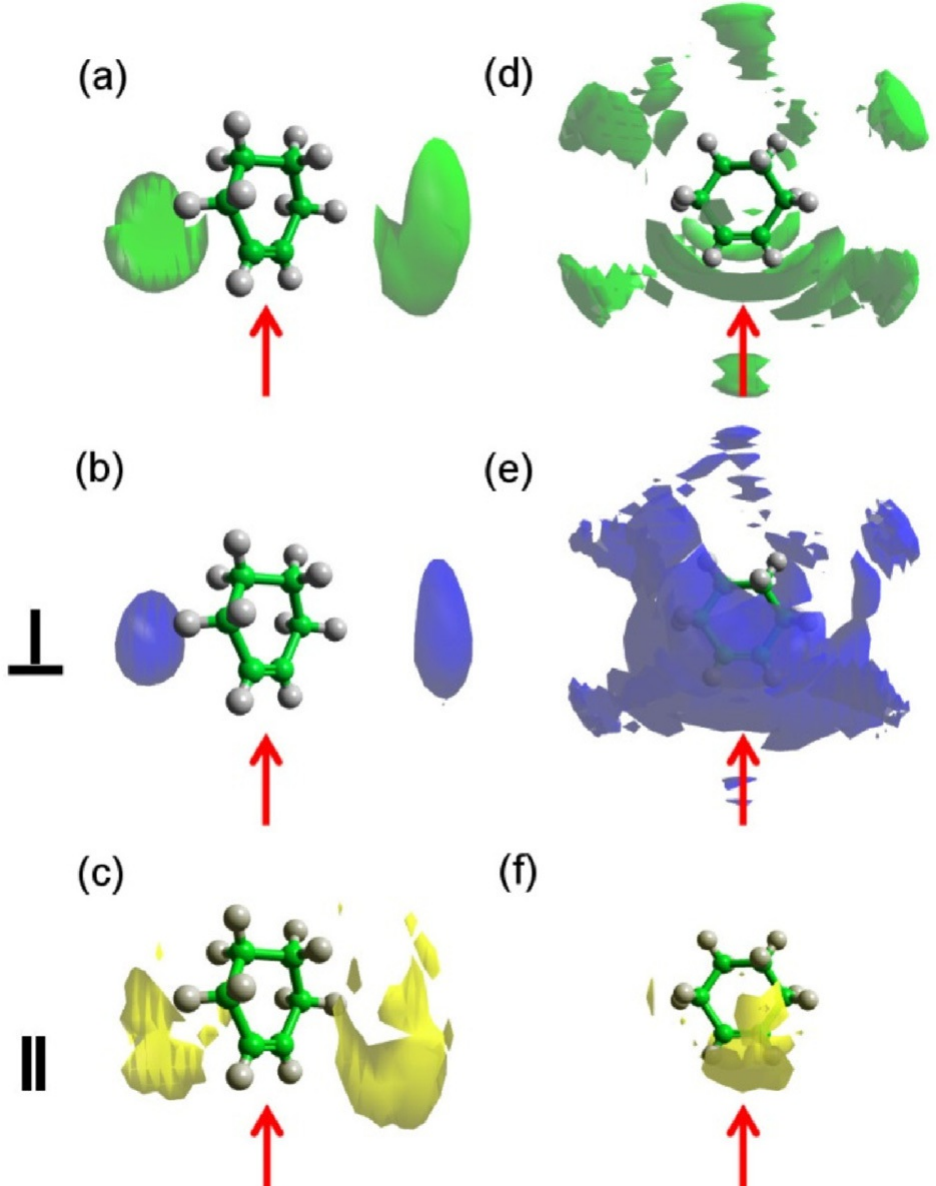
Spatial probability densities for liquid cyclohexene calculated within two distance ranges determined from the ARDF, that is 0–5.25 Å and 5.25–8.10 Å from the central molecule. The functions represent the top 20 % of a) all molecules, b) perpendicular molecules only (*θ*=90±10°) and c) parallel molecules only (*θ*=0±10°) with respect to the central molecule found within *r*=0–5.25 Å. Additionally, functions representing the top 10 % of d) all molecules, e) perpendicular molecules only (*θ*=90±10°) and f) parallel molecules only (*θ*=0±10°) with respect to the central molecule found within *r*=5.25–8.10 Å are shown. Red arrow indicates the position of double bond in the molecule.

Spatial probability density plots for cyclohexene are presented in Figure 8 and show that, at shorter distances (0–5.25 Å), molecules occupy positions below and above the ring. Moreover, a preference for parallel‐oriented molecules to be directly above and below the double bond can be observed, which is not found for perpendicular molecules and is often referred to as parallel displacement. Additionally, the parallel molecules approach the central molecule more closely than the perpendicular molecules.

At longer distances (5.25–8.10 Å), a general six‐fold symmetric distribution of molecules with additional positions below and above the double bond can be identified as the most likely positions for surrounding molecules. The positions perpendicular to the middle of the C=C bond are not occupied with as high a probability as the remaining five equatorial lobes. Moreover, the perpendicular molecules are not only stacked below and above the double bond, but also found in the middle of the C−C bonds with the exclusion of positions in front of a double bond in the plane of the molecule. Parallel‐oriented molecules show a similar spatial distribution to those found at shorter distances with the molecules occupying regions above and below the double bond (parallel displacement). Further analysis of the data shows that within a shorter distances range (0–5.25 Å), approximately 3 % of all of the molecules are parallel and 9 % are perpendicular. At longer distances within the first coordination shell (5.25–8.10 Å), approximately 18 % of molecules are perpendicularly oriented to the central molecule, but only 1.4 % are parallel. Therefore, the presented plots discriminating the orientations of molecules reveal only a small fraction of molecules present in the simulation box.

**Figure 9:**
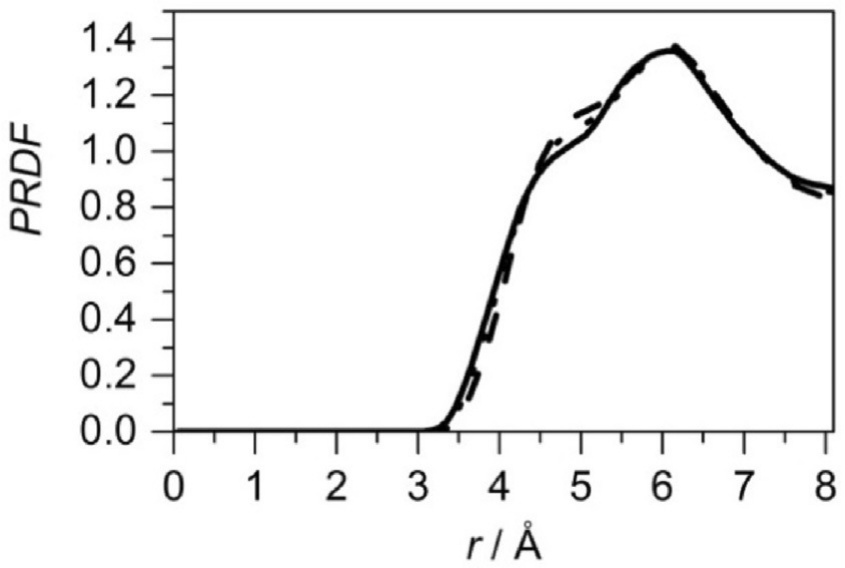
Site–site radial distribution for cyclohexene: the distribution for a double bond around the central molecule's double bond (solid line), the distribution for an adjacent bond (C2−C3 or C1−C6) around the central molecule's double bond (dotted line) and the distribution for an opposite to the double bond (C4−C5) around the central molecule's double bond (dashed line). The distribution of a remaining bond (C3−C4 or C5−C6), next to the C4−C5 bond, around the double bond, shows similar behaviour as the distribution of C4−C5 around the central double bond, and is not shown in the plot for clarity. Each site–site radial distribution was calculated from the middle of the bonds.

To probe the correlations introduced by the presence of the C=C bond, site–site RDFs between the middle of central molecule's double bond and the middle of specific bond in the surrounding molecules (the double bond, C4−C5 bond and C2−C3 bond) were calculated (Figure 9).

As can be seen, the distributions of the middles of the specific bonds around the middle of the double bond of the central molecule have similar shapes. However, a shoulder at shorter distances in the distribution of the C4−C5 bond indicates that it is slightly more probable to find this type of bond than the double bond. This suggests that the double bond prefers to be near the aliphatic end of the molecule, rather than associated with other double bonds in the liquid. However, analysis based on the ARDF and SDF data indicates a preference for double bond–double bond association. Therefore, despite the presence of a smaller number of double bonds in the molecule when compared to other bonds, the strength of the π–π interactions governs the structure of the liquid in this case, as compared with van der Waals interactions.

### 2.3 Methylcyclohexane

For methylcyclohexane, three coordination shells can be distinguished in the simulated RDF (Figure 2). The first coordination shell has its maximum at a separation of 6.5 Å from the central molecule, with a peak value of 2.2 and coordination number of 12.6. The second maximum of the RDF is at 11.4 Å, which is 1.75 times that of the first maximum. The ranges of the coordination shells are similar to these reported by Drozdowski and Nowakowski (i.e. 3.5–8.5 Å, 8.5–13.6 Å, 13.6–18.5 Å).^26^

**Figure 10:**
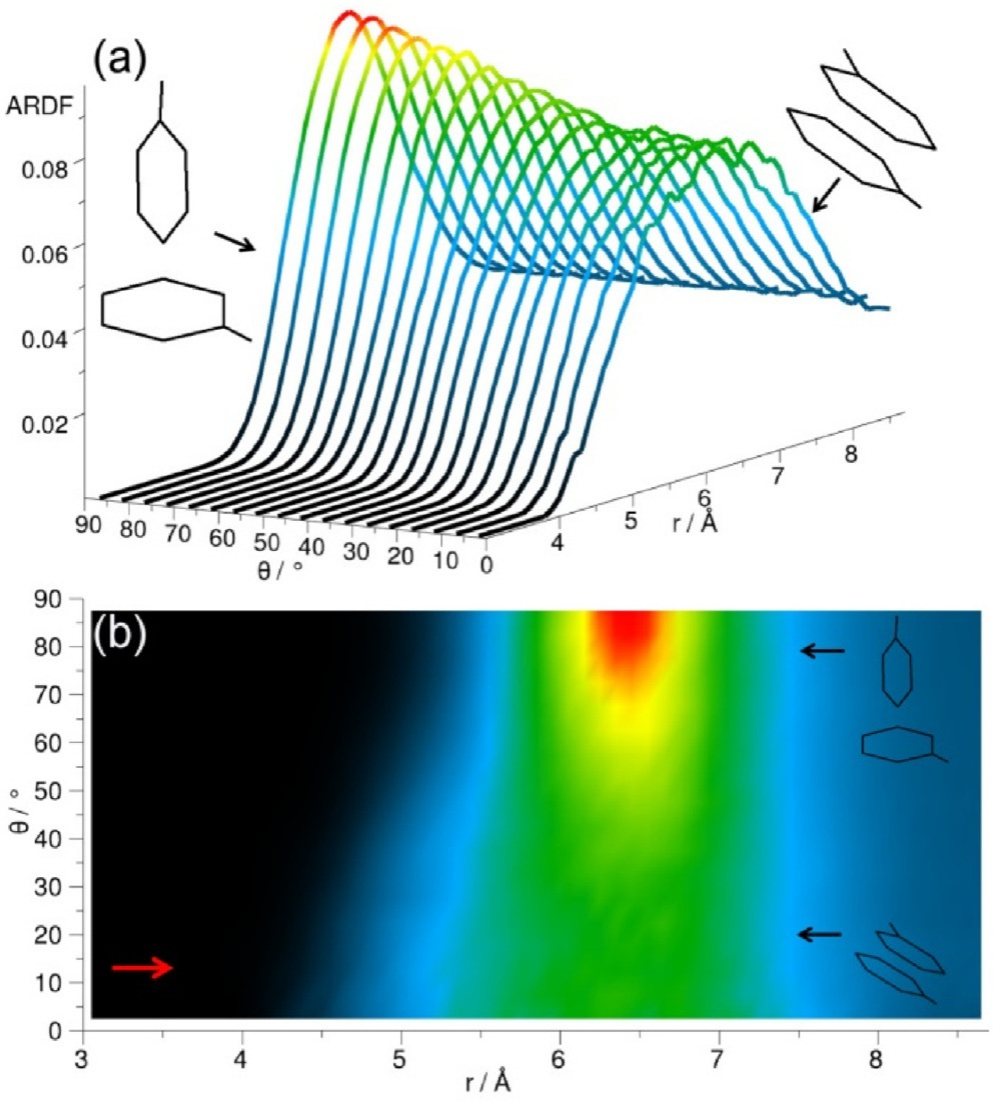
Angular radial distribution function for methylcyclohexane calculated as a function of the angle between the *z* axes of the central and surrounding molecules (0°<*θ*<90°), which in the studied liquid has been defined as shown in Figure 4. Plot (b) is the overhead projection of the 3D plot (a). The red arrow in plot (b) indicates a small increase in ARDF, which signifies a slight preference of the parallel orientation at distances shorter than 5.75 Å.

Clearly, the highest peak in the ARDF for methylcyclohexane within the distance range corresponding to the first RDF coordination shell is observed for the perpendicular arrangement (*θ*=90±10°), but, within this range, parallel‐oriented molecules can also be found (Figure 10). The dominant orientation of molecules in this liquid does not match the findings reported by Drozdowski and Nowakowski,^26^ wherein it was suggested that, in liquid methylcyclohexane, the molecules are arranged with their cyclohexyl rings in parallel. However, at distances shorter than 5.75 Å, a small preference for parallel orientation (*θ*=0±10°) can be found, as shown by the small shoulder (indicated by an arrow in Figure 10 b).

**Figure 11:**
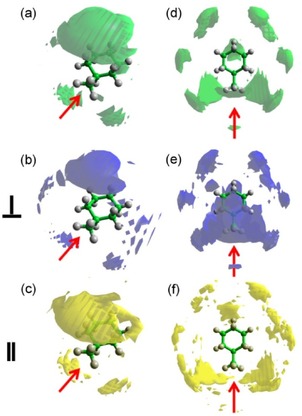
Spatial probability densities for liquid methylcyclohexane calculated within two distances ranges determined from the ARDF, that is, 0–5.75 Å and 5.75–8.70 Å from the central molecule. The functions represent the top 20 % of a) all molecules, b) perpendicular molecules only (*θ*=90±10°) and c) parallel molecules only (*θ*=0±10°) with respect to the central molecule found within *r*=0–5.75 Å. Additionally, functions representing the top 10 % of d) all molecules, e) perpendicular molecules only (*θ*=90±10°) and f) parallel molecules only (*θ*=0±10°) with respect to the central molecule found within *r*=5.75–8.70 Å are shown. The red arrow indicates the position of the methyl group in the central molecule.

The spatial probability densities for the two ranges determined from the ARDF, that is, for surrounding molecules separated from the central one within distance ranges of 0–5.75 Å and 5.75–8.70 Å, were calculated and plotted in Figure 11.

The most probable positions for methylcyclohexane molecules at shorter separations (0–5.75 Å) are surrounding the methyl group. The association of methyl groups is clear for parallel and perpendicularly oriented molecules; however, two distinct arrangements are noted. The parallel molecules, which consist of 2.4 % of all molecules within this range, are found in three distinct lobes around the central methyl group, whereas the perpendicular molecules (ca. 12.4 % of all molecules) surround it creating a “halo”. Parallel molecules approach the central molecule more closely than the perpendicular molecules. At longer distances (5.75–8.70 Å), a six‐fold symmetry of the most probable positions is slightly disrupted by the presence of methyl group, but the general preference of occupying the middle of the C−C bonds can be found. Additionally, stacking below and above the ring can be found within the 10 % most probable positions plotted in Figure 11. Parallel molecules do not occupy positions below and above the ring, but are found in the middle of C−C bonds in the plane of the central molecule. It should be highlighted that positions in the region around the C1−C7 bond have a higher probability for being occupied than the other C−C bonds in the central molecule. Perpendicular molecules within a longer distance range tend to occupy the middle of the C−C bonds as well as stacking below and above the ring, with both positions having similar probabilities. In addition, there are more perpendicularly oriented surrounding molecules (18 %) than parallel (1.5 %) in this region. Regardless of the distance or the orientation, positions axial to the methyl group are not occupied with a significant probability.

**Figure 12:**
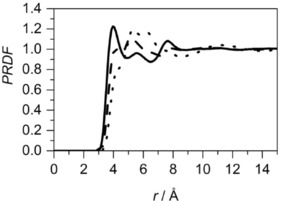
Site–site radial distribution for methylcyclohexane: the distribution for a methyl carbon around the central molecule's methyl carbon (C7 around C7, solid line), the distribution for the carbon opposite the methyl group around the central molecule's methyl carbon (C7 around C4, dashed line) and the distribution for the carbon opposite the methyl group around the central molecule's carbon opposite the methyl group (C4 around C4, dotted line).

To investigate the influence of the methyl group on the local ordering in liquid methylcyclohexane, the site–site PRDFs between the central molecule's methyl carbon (C7) and methyl carbon in the surrounding molecules, as well as between methyl carbon (C7) and the surrounding opposite ends of the molecule (C4) were calculated (Figure 12). In the region separated from the central methyl group of 3.0–4.7 Å, it is more probable to find a methyl group than the C4 carbon, as shown by the increase in the first peak maximum. At longer distances, between 4.7–7.0 Å, the opposite trend is observed with ring–methyl interactions being found, as compared with methyl–methyl interactions. At distances between 7.0–8.7 Å, there is a small enhancement of the methyl–methyl interactions when compared with the ring–methyl interactions, but, at this point, the structure is much less defined, as expected. The association of the methyl groups is also confirmed by examining the site–site PRDF for the carbon opposite the methyl group (C4) in the central and surrounding molecules. This shows similar behaviour to the distribution of the ring–methyl interactions and the reverse trend from the methyl–methyl interactions.

**Figure 13:**
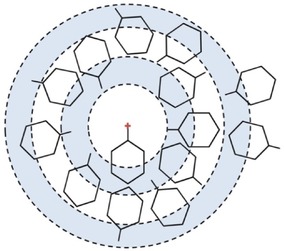
Exemplar motif for methylcyclohexane molecules produced based on the partial distribution functions from Figure 12. It shows the surplus of methyl carbons (C7) when compared with amount of C4 within the distance 3.0–4.7 Å. Within the next shell (4.7–7.0 Å), fewer methyl carbons are present, because fewer of them are available; however, in the next shell (7.0–8.7 Å), more methyl carbons are found.

An exemplar motif of methylcyclohexane molecules is shown in Figure 13. Moreover, it seems that the methyl group has an influence on the local ordering, only within a distance corresponding to the first coordination shell in the centre of geometry RDF.

### 2.4 Benzene

For benzene, three coordination shells are clearly distinguished (Figure 2), which is consistent with previously reported studies.^12^, ^39^ The shape of the primary peak in the RDF has a shoulder at shorter distances, as was also observed in Ref. 12, and which is attributed to parallel π–π stacking of molecules. The maximum of the first coordination shell is at a distance of 5.9 Å from the central molecule and has a peak value of 2.1, and this feature has a coordination number of 13.1. The second maximum is at 10.3 Å, which is 1.75 times that of the first. Headen et al.^12^ reported the first maximum to be at 5.75 Å, and Katayama et al.^32^ found that it was at 6.35 Å. The RDF obtained from molecular dynamic simulations^31^ had a maximum at approximately 5.0 Å. Cabaço et al.^39^ reported the first maximum to be at 5.5 Å, and the second to be at 10.0 Å, which is in good agreement with our findings.

**Figure 14:**
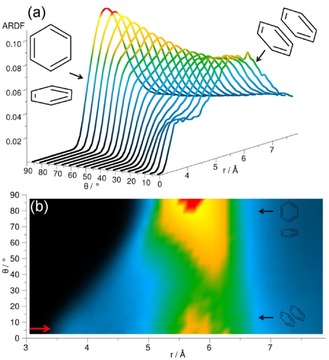
Angular radial distribution function for benzene calculated as a function of the angle between the *z* axes of the central and surrounding molecules (0°<*θ*<90°), which in the studied liquid has been defined as shown in Figure 4. Plot (b) is the overhead projection of the 3D plot (a). The red arrow in plot (b) indicates an increase in ARDF, which signifies a slight preference of the parallel orientation at distances shorter than 4.85 Å.

To investigate the preferable orientations of molecules with respect to each other, the ARDF for benzene was calculated (Figure 14). At distances shorter than 4.85 Å, the molecules tend to have a parallel orientation; whereas, at longer distances, perpendicular orientation is preferred (although parallel geometries are also present). The same tendency is found by Headen et al.,^12^ who identified the cut‐off to be at 5.0 Å. The model used by Baker and Grant^28^ indicated that the structure in the first coordination shell is mainly perpendicular. Similarly, Katayama et al.^32^ stated that the parallel configuration is not dominant, and that more perpendicular molecules with respect to the central molecule can be found within the first association shell. Cabaço et al.^39^ found no specific orientations for surrounding molecules at distances greater than 5.0 Å, but they also highlighted the preference for parallel arrangement at distances shorter than 4.5 Å, owing to better packing.

**Figure 15:**
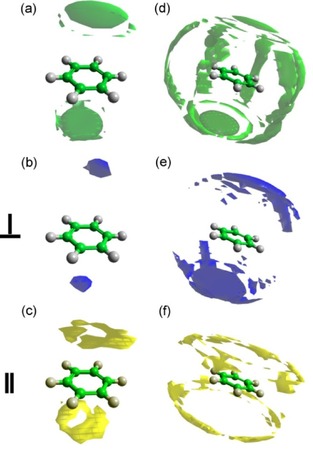
Spatial probability densities for liquid benzene calculated within two distance ranges determined from the ARDF, that is, 0–4.85 Å and 4.85–7.90 Å from the central molecule. The functions represent the top 20 % of a) all molecules, b) perpendicular molecules only (*θ*=90±10°) and c) parallel molecules only (*θ*=0±10°) with respect to the central molecule found within *r*=0–4.85 Å. Additionally, functions representing the top 10 % of d) all molecules, e) perpendicular molecules only (*θ*=90±10°) and f) parallel molecules only (*θ*=0±10°) with respect to the central molecule found within *r*=4.85–7.90 Å are shown.

Spatial probability density plots for benzene (Figure 15) show that molecules at shorter distances prefer to occupy positions parallel to the ring, with the parallel‐oriented molecules being closer to the central molecule than those in a perpen dicular orientation. Additionally, they do not occupy positions precisely above and below the middle of the central molecules ring (parallel displacement), whereas the perpendicular molecules fill these positions. At distances shorter than 4.85 Å, 4.6 % of all molecules are parallel and 2.9 % are perpendicular. At longer distances (4.85–7.90 Å), molecules occupy positions in the middle of the C−C bonds, in general, as well as above and below the ring. The parallel‐oriented surrounding molecules create two halos above and below the central molecule's ring, causing an arrangement of a parallel displacement, which was also reported in the previous study.^12^ The most probable positions for perpendicularly oriented molecules are above and below the ring. The previous neutron diffraction study^12^ showed a similar tendency for surrounding molecules to that presented here for distances longer than 4.85 Å. However, no investigation of shorter distances was conducted. In the present work, the investigated range contained approximately 1.6 % of all molecules that are in a parallel orientation and 18.8 % in a perpendicular orientation.

**Figure 16:**
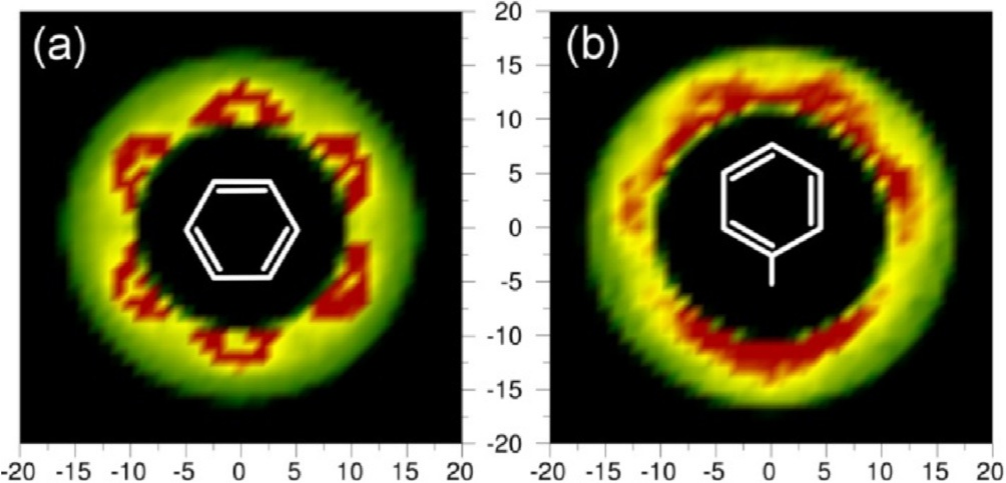
Two‐dimensional cuts through the spatial probability density function for perpendicularly oriented molecules within the specified distance range for: a) benzene 4.85–7.90 Å and b) toluene 5.3–8.3 Å. Red colour indicates the highest probability of the position to be occupied by perpendicular molecules to the central molecule [at position (0,0)].

The perpendicularly oriented surrounding molecules may be positioned with respect to the central molecule in one of two significant arrangements: either one (T‐shape) or two adjacent (Y‐shape) H atoms of the central molecule point toward the surrounding molecule. To determine the type of interactions between perpendicular molecules, a two‐dimensional cut through the spatial density function for all of the perpendicular molecules present between 4.85 and 7.9 Å was taken and is shown in Figure 16 a. The red colour indicates the most probable positions of surrounding perpendicular molecules around the central molecule, which is in the plane of the plot. These are found for surrounding molecules that are axial to the middle of C−C bonds in the central molecule, which corresponds to the Y‐shape arrangement, which is in agreement with the previous study.^12^

### 2.5 Toluene

For toluene, two broad coordination shells in the RDF can be observed, in agreement with previous reports,^12^, ^31^, ^34^ which suggests that, within each coordination shell, molecules have a few preferable orientations to occupy (which differ by the separation between the central and surrounding molecules), as shown in Figure 2. The first coordination shell maximum is found at a distance of 6.0 Å with a peak value of 1.6. The number of molecules found within the first coordination shell is 13.3. The second maximum in the RDF is found 1.80 times further out in *r* than the first, at a distance of 10.8 Å. The local ordering in liquid toluene investigated by using X‐ray diffraction^32^ also shows a broad peak in the centre of geometry RDF at a similar distance to that found in this work (at 6.0 Å). A molecular dynamics study of liquid toluene^34^ also confirms the position of the first coordination shell at 6.1 Å with 12 molecules, and the second maximum at 10 Å.

**Figure 17:**
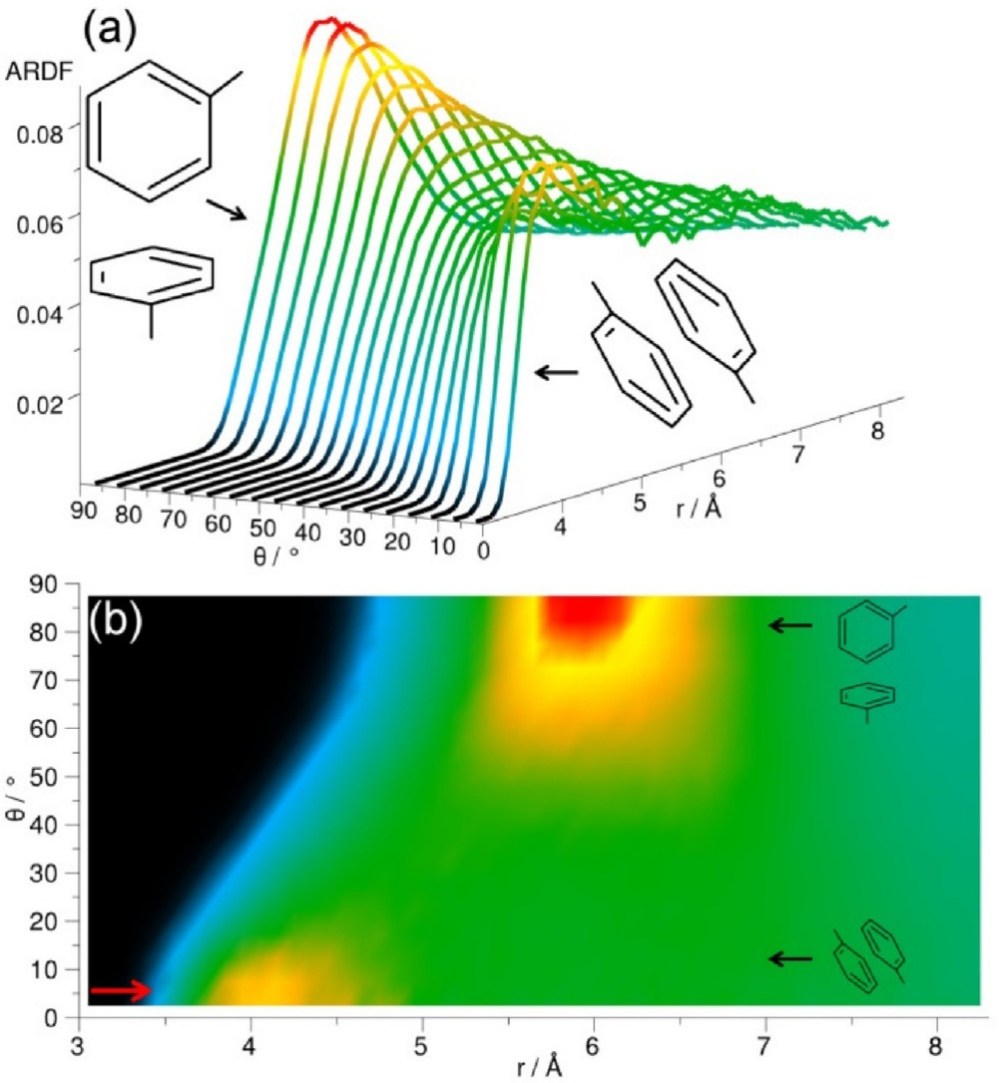
Angular radial distribution function for toluene calculated as a function of the angle between the *z* axes of the central and surrounding molecules (0°<*θ*<90°), which in the studied liquid has been defined as shown in Figure 4. Plot (b) is the overhead projection of the 3D plot (a). The red arrow in plot (b) indicates an increase in ARDF, which signifies a slight preference of the parallel orientation at distances shorter than 5.3 Å.

Figure 17 shows the ARDF for toluene. Molecules separated from the central molecule by more than 5.3 Å are mostly perpendicular (global maximum at 6.0 Å); whereas, at distances shorter than 5.3 Å they tend to be in a parallel orientation with a peak visible at local maximum at a distance of 4.0 Å. The study conducted by Headen et al.^12^ reveals a similar preference for the parallel orientation of surrounding molecules at distances shorter than 5.0 Å and the preference for perpendicular at longer separations; however, the former are said to be dominant in the whole first coordination shell. Molecular dynamic studies^34^ show a prevalence of the parallel stacking between dimers of toluene molecules at a distance of 3.1 Å from the centre of the ring of the central molecule. At longer distances, the lack of specific orientation after the first neighbour is suggested. Monte Carlo simulations^35^ showed equal preference for parallel and perpendicular orientations at shorter distances.

**Figure 18:**
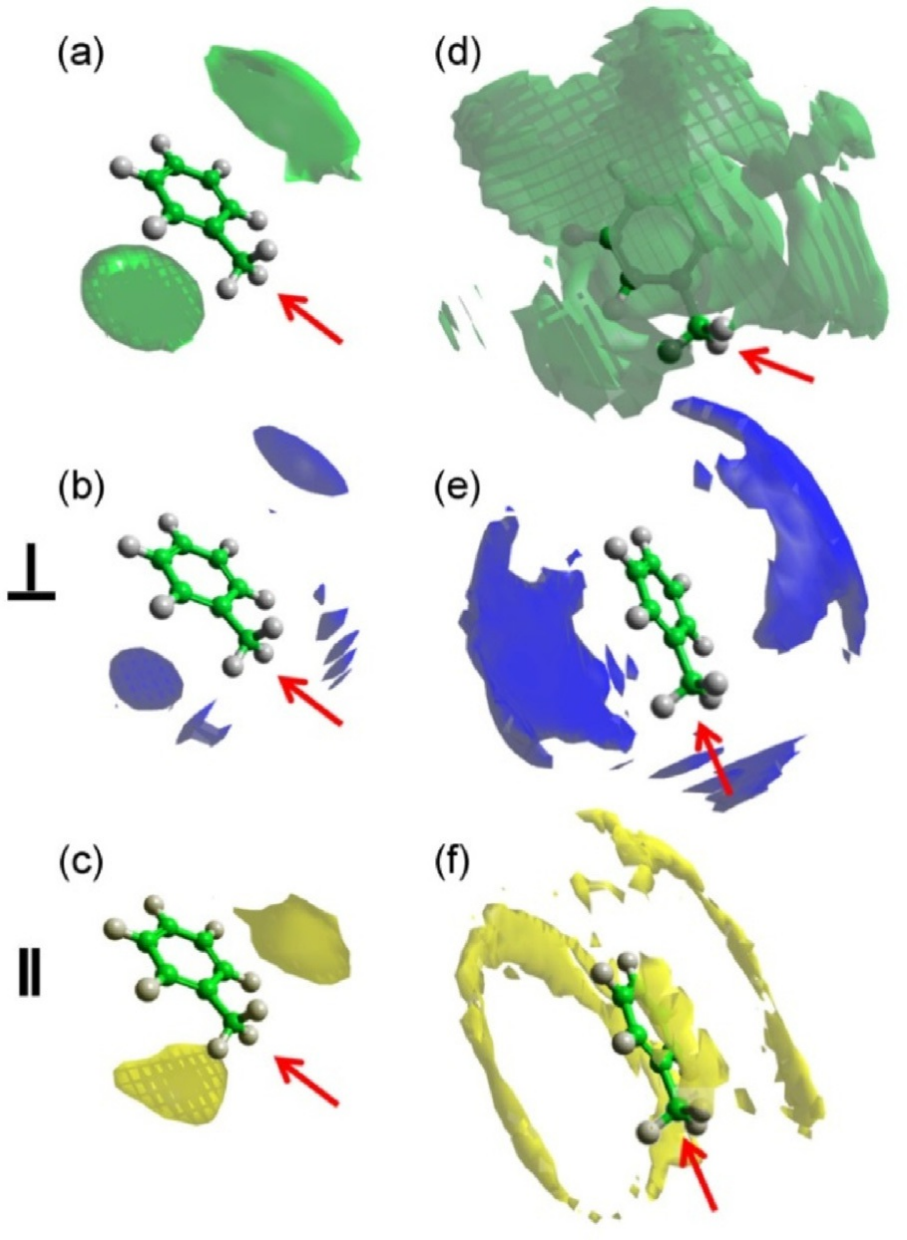
Spatial probability densities for liquid toluene calculated within two distance ranges determined from the ARDF, that is, 0–5.3 Å and 5.3–8.3 Å from the central molecule. The functions represent the top 20 % of a) all molecules, b) perpendicular molecules only (*θ*=90±10 and c) parallel molecules only (*θ*=0±10°) with respect to the central molecule found within *r*=0–5.3 Å. Additionally, functions representing the top 10 % of d) all molecules, e) perpendicular molecules only (*θ*=90±10°) and f) parallel molecules only (*θ*=0±10°) with respect to the central molecule found within *r*=5.3–8.3 Å are shown. The red arrow indicates the position of the methyl group in the central molecule.

From the calculated spatial probability density functions (Figure 18), at distances shorter than 5.3 Å, toluene molecules prefer to occupy positions parallel to the ring, but shifted slightly away from the middle of the ring towards the methyl group (parallel displacement). The parallel molecules within this range show the preference to occupy positions above and below the methyl group (parallel displacement), whereas perpendicular molecules place themselves above and below the ring as well as around the methyl group. At distances longer than 5.3 Å, molecules occupy positions in the middle of C−C bonds, but avoid being axial to the methyl group. Parallel‐oriented molecules show preferential positions that create halos above and below the toluene ring (parallel displacement), but do not interact with the methyl group. In contrast, whilst the perpendicular molecules are occupying the positions above and below the ring, they also are found axial to the methyl group. The lobes for both orientations confirm that the parallel can approach the central molecule more closely than those with a perpendicular orientation. At short distances (0–5.3 Å), the ratio between perpendicular‐ and parallel‐oriented molecules is 2.5:1; whereas, at longer distances, the perpendicular molecules are significantly more dominant with a ratio of 12.8:1. The equally well‐populated parallel and perpendicular arrangements were found by Baker and Grant,^35^ who studied toluene by using Monte Carlo simulations with two different force fields.

The perpendicular molecules in toluene show the Y‐shape arrangement around the ring at the aromatic end of the molecule (Figure 16 b), which was also found in the previous study.^12^ Additionally, the high probability is found for the positions directed axially towards the central molecule's methyl group. In this case, the centres of geometry of the surrounding perpendicular molecules are pointed with three H atoms from the methyl group in the central molecule. In contrast to our findings, the studies conducted based on Monte Carlo simulations^35^ show a slight preference towards the T‐shaped structure.

**Figure 19:**
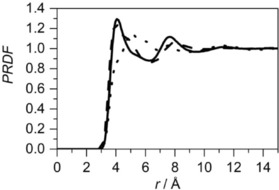
Site–site radial distribution functions for toluene: the distribution for a methyl carbon around the central molecule's methyl carbon (C7 around C7, solid line), the distribution for the carbon opposite the methyl group around the central molecule's methyl carbon (C7 around C4, dashed line) and the distribution for the carbon opposite the methyl group around the central molecule's carbon opposite the methyl group (C4 around C4, dotted line).

The site–site PRDF between the central molecule's methyl carbon (C7) and methyl carbon (C7) in the surrounding molecules, as well as between the methyl carbon (C7) and the surrounding opposite ends of the molecule, across the ring (C4) are shown in Figure 19. Both PRDFs have the first maximum at the same distance, and these peaks also have similar intensities, indicating that both interactions are equally probable. The second shell is found between 6.4 and 9.5 Å from the central methyl group. The length of the toluene molecule (distance between C7 and C4) is 4.23 Å and is almost identical to the separation between two peaks in the PRDFs. This suggests that local ordering is governed by the presence of the methyl groups; however, it is evident only within the distance corresponding to the first coordination shell in centre of geometry—centre of geometry RDF. Additionally, the function between the carbon opposite the methyl group (C4) on the central and surrounding molecules was calculated, and it reveals the reverse trend as presented in both PRDFs described above. Similar shapes and positions of the peaks (at 4.1 and 7.7 Å) in the site–site RDFs between methyl carbon in the central and surrounding molecules were found by Fioroni and Vogt^34^ from the molecular dynamics study of toluene structure. Monte Carlo simulations^35^ confirm the same association of methyl groups at a distance of approximately 4.1 Å and neutron diffraction studies highlight the first maximum at 4.14 Å.^12^

### 2.6 Comparison of Local Ordering in C6 Hydrocarbons

All of the five investigated liquids have similar intramolecular interactions, as the total structure factor functions are almost superimposable in the 5–20 Å^−1^ range (Figure 1). The intermolecular interactions tend to occur at smaller distances in aromatic liquids than in aliphatic liquids, as shown by the shift in the first shell maximum in Figure 2, which indicates stronger molecule–molecule bonding in the aromatic systems. This shift is most prominent for benzene. The influence of the double bond in the structure is confirmed by the differences in the centre of geometry RDF peaks and the more well‐defined positions for cyclohexane and cyclohexene. In methylcyclohexane and toluene, the presence of a methyl group in the structure causes a shift of the first coordination shell to longer separations from the central molecule when compared with cyclohexane and benzene, respectively. This is expected from the increase in the molecular volume. However, the π–π interactions on the intermolecular approach a distance for the surrounding molecules that is more structure determining than the methyl interactions, as a smaller shift is observed between toluene and benzene compared with between methylcyclohexane and cyclohexane.

The second shell maxima are found to be approximately 1.75–1.80 times that of the distance for the first coordination shell maxima for all investigated liquids, which can be attributed to the packing phenomena. It is noticeable that the peaks in RDF are highest in cyclohexane, where there is no disruption in the local ordering, owing to the introduction of a methyl group or a double bond. In toluene, the presence of both the methyl group and the aromatic ring has an influence on the liquid structure. In this case, the peaks are much broader and the peak maxima are lower, which may suggest anisotropic behaviour of the surrounding molecules in this liquid caused by a steric effect.

Interestingly, all of the liquids studied herein have similar first shell coordination numbers, between 12.6–13.4 molecules. Benzene has a slightly increased population in the first shell relative to the aliphatic compounds, which may be driven by the fact that the molecules are flat and can subsequently pack more efficiently than in cyclohexane derivatives. In toluene, the presence of the methyl group leads to a marked decrease in the first shell coordination number when compared to benzene, which may again be attributed to a decrease in the ability to pack.

The analysis of the ARDFs shows that, in all of the liquids, two regions within the first coordination shell in the RDFs are found with different preferable orientations. At shorter distances, more molecules are found to be parallel than at longer distances (Table 2); but, with the exception of benzene, the perpendicular orientations dominate at shorter separations from the central molecule. In benzene, at shorter distances, more parallel molecules are found compared to those with a perpendicular orientation.

**Table 2:** Populations of parallel and perpendicular molecules of all molecules present in the specific range. For each liquid, the shorter and longer distances are determined from the corresponding ARDFs.

Compound	Parallel molecules [%]		Perpendicular molecules [%]	
	Shorter distances	Longer distances	Shorter distances	Longer distances
cyclohexane	2.3	1.5	12.3	17.8
cyclohexene	3.2	1.4	9.2	17.9
methylcyclohexane	2.4	1.5	12.4	18.0
benzene	4.6	1.6	2.9	18.1
toluene	3.6	1.4	8.8	18.4

At longer distances, perpendicular molecules always have a larger population, with almost 20 % of all molecules found to occupy this geometry in all cases. In contrast, parallel molecules make up only 1.5 % of all molecules at longer separations from the central molecule. Additionally, the number of all specifically oriented molecules (i.e. parallel or perpendicular) account for only a small portion (<22 %) of the total number of molecules in the first coordination shell in the centre of geometry RDFs. This emphasises the fact that, although specific configurations may be important for the chemical behaviour of these liquids, most of the molecules have generally disordered intermolecular interactions.

The spatial probability functions calculated for all liquids within two distance ranges from the central molecule determined from the ARDF for each liquid show different tendencies occurring in each region.

For all liquids, SDFs at shorter distances show that the surrounding molecules prefer to stack above and below the ring. The presence of the double bond in the structure causes a shift in the preferred positions for cyclohexene molecules to be above and below this bond; whereas, in benzene, these positions are shifted back to the middle of the ring, as expected from C6 symmetry. The influence of methyl group on the preferable positions at shorter distances leads to lobes present around the methyl group in both methylcyclohexane and toluene, predominantly as a result of methyl–methyl interactions.

Parallel molecules at shorter distances are found closer to the central molecule than the perpendicular molecules, as expected from the packing efficiency in all liquids. In cyclohexane, cyclohexene, benzene and toluene, perpendicularly oriented molecules at shorter distances are placed directly above and below the ring; however, in toluene, additional density is found around the methyl group. These positions are strongly occupied by perpendicular molecules in methylcyclohexane, where no stacking below and above the ring is present. Parallel molecules within shorter distances in cyclohexene occupy positions shifted to above and below the double bond (parallel displacement) as compared to cyclohexane (sandwich arrangement), and similar behaviour can be observed for toluene, where the parallel oriented molecules place themselves above and below the methyl group. A different distribution is found in methylcyclohexane, where parallel molecules surround the methyl group, and in benzene, where no density is found directly above and below the middle of the central molecule ring (parallel displacement).

At longer distances within the first coordination shell in the centre of geometry RDFs, there is a general tendency to occupy positions perpendicular to the middle of the C−C bonds. Additionally, stacking below and above the double bond for cyclohexene and above and below the ring for benzene are present. In methylcyclohexane and toluene, positions below and above the ring are also occupied, and this is more prominent for the aromatic molecule compared with the aliphatic. In both liquids, little density is found axial to the methyl group.

Perpendicular molecules at longer ranges within the first coordination shell in the centre of geometry RDFs for cyclohexane and methylcyclohexane show a six‐fold symmetric density distribution around the ring with, in the latter case, additional density above and below the ring. In cyclohexene, stacking above and below the double bond by perpendicular molecules is evident, but also density at positions in the middle of single bonds is observed. In benzene and toluene, similar stacking, but below and above the middle of the ring, is observed. Additionally, for liquids with methyl groups in their structure, density is found axial to the methyl group.

Parallel molecules at longer distances from the central molecule create a halo of density above and below the ring for benzene and toluene and stacking below and above the double bond in cyclohexene. In contrast, in cyclohexane and methylcyclohexane, there is a preference for positions perpendicular to the middle of C−C bonds. In both liquids, the parallel molecules do not approach the central molecule as closely as in the others, because of the H atoms pointing out of the ring, which has a repelling influence on the surrounding molecules. Additionally, the lack of delocalised π electrons in the structure weakens the interactions between molecules. In general, parallel molecules at longer distances within the first coordination shell in centre of geometry RDFs do not show preference for a sandwich arrangement in any of the studied liquids.

**Figure 20:**
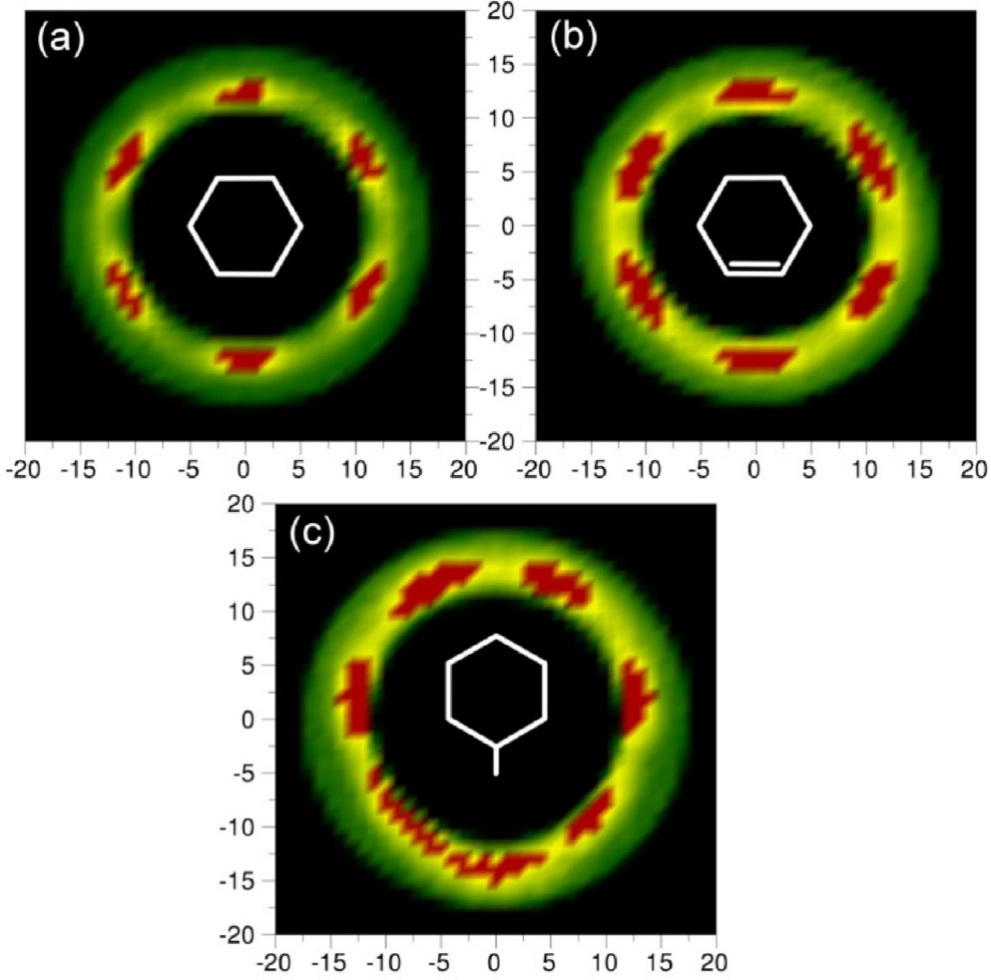
Two‐dimensional cuts through the spatial probability density function for perpendicularly oriented molecules within the specified distance range for: a) cyclohexane 5.45–8.30 Å, b) cyclohexene 5.25–8.10 Å and c) methylcyclohexane 5.75–8.70 Å. Red colour indicates the highest probability of the position to be occupied by perpendicular molecules to the central molecule [at position (0,0)].

As shown in Figure 16 a, perpendicular surrounding benzene molecules have a tendency to be in the Y‐shape arrangement around the central molecule's ring. The same trend of filling positions in the middle of central molecule's C−C bonds (two H atoms pointing to the geometric centre of a surrounding molecule) by perpendicular surrounding molecules around the ring is observed for toluene. Additionally, the geometric centres of the surrounding perpendicular molecules tend to associate with the methyl group of the central molecule. For aliphatic liquids, identical behaviour is found (Figure 20), that is, perpendicular molecules show the Y‐shape trend axially around the central molecule's ring, and the presence of a methyl group causes the same disruption in the arrangement as observed for toluene. This suggests that aromaticity does not influence the arrangement of perpendicular molecules around the central molecule's ring; however, the presence of a methyl group does appear to be structure determining.

**Figure 21:**
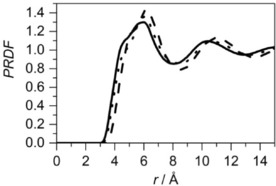
Site–site radial distribution functions between specific bonds: cyclohexane single bond (dashed line), cyclohexene double bond (dotted line) and benzene double bond (solid line). Each site–site distribution was calculated from the middle of the specific bonds.

The partial site–site RDFs calculated for cyclohexene show that the double bond has a preference to be near the aliphatic end of the molecule, rather than associated with the double bond. The comparison of distributions of a single bond in cyclohexane, a chosen bond in benzene and double bond in cyclohexene around the corresponding bonds in the central molecule is shown in Figure 21. This shows that, with increasing number of double bonds in the molecular structure, there is a decrease in the distance between the molecules. The integration up to the first minimum in each RDF shows that, for cyclohexane, slightly fewer bonds are present within the first association shell (13.8) than in cyclohexene (14.3) and benzene (14.1). It seems that the presence of the double bond in the structure does not have a significant influence on the association, but the unsaturated bond interactions are stronger.

**Figure 22:**
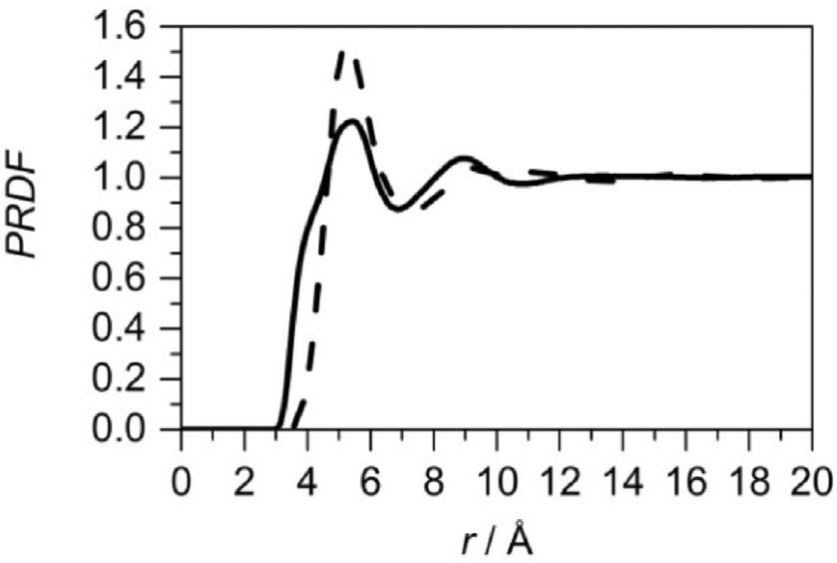
Site–site radial distribution function for methyl carbons (C7) in surrounding molecules around the centre of geometry of central molecule for liquid methylcyclohexane (dashed line) and toluene (solid line).

Analysis of partial RDFs for aromatic and aliphatic molecules with methyl groups present in the structure shows the different type of methyl group association. In methylcyclohexane, the surplus of methyl groups to the opposite end of the molecule can be observed, whereas for toluene they are equally present within the first association shell. Additionally, in methylcyclohexane, three different environments can be found, whereas there are only two for toluene. This directly indicates the influence of the presence of delocalised π electrons in the structure, which strongly organise it. Further analysis of this can be achieved by comparing the site–site RDFs of methyl carbons in surrounding molecules around the centre of geometry of central molecule for both liquids (Figure 22). Clearly, methyl groups from surrounding molecules approach the centre of the geometry of the central toluene more closely than the central methylcyclohexane. Nevertheless, more functional groups can be found within the first association shell in the aliphatic compound (7.5) when compared to an aromatic compound (6.8).

## 3 Conclusions

Five liquids, that is, cyclohexane, cyclohexene, methylcyclohexane, benzene and toluene, have been studied by neutron diffraction measurements to establish the influence of the presence of double bonds and methyl groups in a molecule on the local ordering.

The discussion of the positions of maxima in radial distribution functions of the centre of geometry of molecules, their intensities and corresponding coordination numbers showed differences in the intermolecular interactions for all liquids. The angular radial distribution functions have been calculated to provide information on the preferable orientations with respect to the central molecule. Two regions within the first coordination shell, in which surrounding molecules show different behaviour in approaching the central molecule, were distinguished for each liquid. The influence of the presence of a double bond in the structure has been investigated by analysing the partial radial distribution functions for a double bond, opposite to a double bond and adjacent to a double bond in surrounding molecules to a double bond in the central molecule. Additionally, the distributions of chosen bonds in cyclohexane, cyclohexene and benzene were compared. The presence of the double bond has an influence on the separation that surrounding molecules can achieve rather than causes bond association.

The influence of the presence of methyl group in the structure has been investigated by analysing partial radial distribution functions for methyl carbon and the opposite end of the molecule of the surrounding molecules around the methyl carbon in the central molecule. Additionally, the distributions of methyl group in surrounding molecules around centre of geometry for methylcyclohexane and toluene were compared. The presence of the methyl group results in their association. However, when both a methyl group and delocalised π electrons are present, the latter has a stronger influence on the local ordering in the liquid.

## Supporting information

As a service to our authors and readers, this journal provides supporting information supplied by the authors. Such materials are peer reviewed and may be re‐organized for online delivery, but are not copy‐edited or typeset. Technical support issues arising from supporting information (other than missing files) should be addressed to the authors.

Supplementary
