# Supplementary material for: Neutron Scattering of Aromatic and Aliphatic Liquids
Source: Chemphyschem. 2016 Apr 13;17(13):2043–55. doi: 10.1002/cphc.201600149 (PMC4999024; doi:10.1002/cphc.201600149)
Supplement: Supplementary file 1 — Supplementary [file CPHC-17-2043-s001.pdf]

CHEMPHYSCHEM

Supporting Information

Neutron Scattering of Aromatic and Aliphatic Liquids

Marta Falkowska,[a, b] Daniel T. Bowron,[a] Haresh G. Manyar,[b] Christopher Hardacre,*[b] and Tristan G. A. Youngs*[a]

cphc_201600149_sm_miscellaneous_information.pdf

Experimental Section

All neutron diffraction data were collected using the Near and InterMediate Range Order Diffractometer (NIMROD)[1] at the ISIS pulsed neutron and muon source at the Rutherford Appleton Laboratory, UK. NIMROD was designed to allow comprehensive studies, ranging over atomic to mesoscopic length scales, of liquids and other disordered materials in a single measurement. The instrument makes use of neutrons of wavelengths from 0.05 to 14 Å which are resolved using a time-of-flight method and is, consequently, able to collect data over a Q-range of 0.01 to 50 $Å^{-1}$.

For each pure liquid, i.e. cyclohexane, cyclohexene, methylcyclohexane, benzene and toluene, scattering data on the fully protiated and fully deuteriated compounds as well as their equimolar mixture were collected to take full advantage of the isotopic contrast between hydrogen and deuterium. All chemicals were purchased from Sigma Aldrich, except toluene-d_8, cyclohexene-d_{10}, and methylcyclohexane-d_{14} which were purchased from Qmx and used without further purification. The liquids were loaded into null-scattering $Ti_{0.68}Zr_{0.32}$ flat plate containers of internal dimensions 35 × 40 × 1 mm with a wall thickness of 1 mm. The cells were sealed with a PTFE O-ring and placed on an automatic sample changer. During the measurements, the samples were maintained at a temperature of 298 $\pm$ 0.1 K using a recirculating water bath to control the temperature of the cell mounting frame. Additional measurements were made on each of the empty sample cells, the empty diffractometer and a 3.0 mm thick vanadium standard sample. Data were corrected for multiple scattering and absorption, and normalized to the incoherent scattering of vanadium using the Gudrun software.[2] Residual inelasticity effects arising from the presence of hydrogen were removed using the iterative procedures in.[2]

The corrected neutron diffraction data were subsequently analysed using the Empirical Potential Structure Refinement (EPSR) method which facilitates the construction of three dimensional atomistic structural models of liquid and disordered material samples that are consistent with the experimental scattering data.[4] Briefly, a Monte Carlo simulation of a representative box of molecules is performed using a reference interatomic potential. From this simulation, the structure factors corresponding to those isotopic systems measured experimentally can be calculated and compared, and a perturbing potential (the empirical potential) can be constructed and applied to the Monte Carlo simulation. This procedure has the effect of driving the structure of the simulation box toward agreement with the experimental data. Once good agreement between simulated and experimental structure factors was obtained, the properties of interest such as site–site radial distribution functions, angular distribution functions and spatial probability densities may be derived from the resulting model. For each neat liquid the EPSR refinement was initialized from an equilibrated Monte Carlo simulation at 298 K containing 400 molecules in a cubic box and the corresponding atomic density derived from the experimentally determined molecular densities of the fully protiated liquids presented in Table S1. Reference potential parameters for each pure liquid were taken from the OPLS-AA force field,[3] Table S2. Structural properties were calculated using a combination of built-in routines in EPSR[4] and custom analysis codes.[5]

Table S1. Simulation box sizes and densities

Compound	Density / atoms $Å^{-3}$	Box length / Å
Cyclohexane	0.0997	41.64
Cyclohexene	0.0951	40.67
Methylcyclohexane	0.0992	43.92
Benzene	0.0809	39.01
Toluene	0.0848	41.36

Table S2. Lennard-Jones potentials and charges used in Empirical Potential Structure Refinement (EPSR) simulations for cyclohexane, cyclohexene, methylcyclohexane, benzene and toluene.

Atom	Description	ε/kJ mol^{-1}	σ/Å	q/e
Cyclohexane				
CA	C	0.276	3.500	-0.120
HA	H	0.126	2.500	0.060
Cyclohexene				
CA	Double-bond-C	0.318	3.550	-0.115
CB	C	0.276	3.500	-0.120
HA	H	0.126	2.500	0.060
HB	Double-bond-H	0.126	2.420	0.115
Methylcyclohexane				
CA	Junction-C	0.276	3.500	-0.060
CB	Ring-C	0.276	3.500	-0.120
CC	Methyl-C	0.276	3.500	-0.180
HA	H	0.126	2.500	0.060
Benzene				
CA	C	0.293	3.550	-0.115
HA	H	0.126	2.420	0.115
Toluene				
CA	Junction-C	0.318	3.550	-0.115
CB	Ring-C	0.293	3.550	-0.115
CC	Methyl-C	0.276	3.550	-0.065
HA	Ring-H	0.126	2.420	0.115
HB	Methyl-H	0.126	2.500	0.060

[1] D. T. Bowron, A. K. Soper, K. Jones, S. Ansell, S. Birch, J. Norris, L. Perrott, D. Riedel, N. J. Rhodes, S. R. Wakefield, A. Botti, M. A. Ricci, F. Grazzi, M. Zoppi *Rev. Sci. Instrum*. **2010**, *81*, 033905, DOI: 10.1063/1.3331655

[2] A. K. Soper, GudrunN and GudrunX: Programs for Correcting Raw Neutron and X-ray Diffraction Data to Differential Scattering Cross Section. RAL Technical Reports RAL-TR-2011-013, **2011**.

[3] W. L. Jorgensen, J. Tirado-Rives, *J. Am. Chem. Soc.* **1988**, *110*, 1657-1666, DOI: 10.1021/ja00214a001.

[4] A. K. Soper, Empirical Potential Structure Refinement - EPSRshell : a User's Guide. Version 18 - May 2011. RAL Technical Reports RAL-TR-2011-012, **2011**.

[5] T. G. A. Youngs, dlputils, version 1.4.1; http://www.projectaten.net/dlputils (accessed 12 August, 2015)
